# Modulation of *Mycobacterium tuberculosis*-specific humoral immune responses is associated with *Strongyloides stercoralis* co-infection

**DOI:** 10.1371/journal.pntd.0005569

**Published:** 2017-05-01

**Authors:** Rajamanickam Anuradha, Saravanan Munisankar, Yukti Bhootra, Chandrakumar Dolla, Paul Kumaran, Thomas B. Nutman, Subash Babu

**Affiliations:** 1 National Institutes of Health—NIRT—International Center for Excellence in Research, Chennai, India; 2 National Institute for Research in Tuberculosis, Chennai, India; 3 Laboratory of Parasitic Diseases, National Institutes of Allergy and Infectious Diseases, National Institutes of Health, Bethesda, Maryland, United States of America; George Washington University, UNITED STATES

## Abstract

**Background / Objectives:**

Helminth infections are known to influence T cell responses in latent tuberculosis (LTBI). Whether helminth infections also modulate B cell responses in helminth-tuberculosis co-infection is not known.

**Methods:**

We assessed *Mycobacterium tuberculosis* (Mtb)–antigen specific IgM and IgG levels, circulating levels of the B cell growth factors, BAFF and APRIL and the absolute numbers of the various B cell subsets in individuals with LTBI, LTBI with coincident *Strongyloides stercoralis* (Ss) infection (LTBI/Ss) and in those with Ss infection alone (Ss). We also measured the above-mentioned parameters in the LTBI-Ss group after anthelmintic therapy.

**Results:**

Our data reveal that LTBI-Ss exhibit significantly diminished levels of Mtb-specific IgM and IgG, BAFF and APRIL levels in comparison to those with LTBI. Similarly, those with LTBI-Ss had significantly diminished numbers of all B cell subsets (naïve, immature, classical memory, activated memory, atypical memory and plasma cells) compared to those with LTBI. There was a positive correlation between Mtb—antigen specific IgM and IgG levels and BAFF and APRIL levels that were in turn related to the numbers of activated memory B cells, atypical memory B cells and plasma cells. Finally, anthelmintic treatment resulted in significantly increased levels of Mtb—antigen specific IgM and IgG levels and the numbers of each of the B cell subsets.

**Conclusions:**

Our data, therefore, reveal that Ss infection is associated with significant modulation of Mtb-specific antibody responses, the levels of B cell growth factors and the numbers of B cells (and their component subsets).

## Author summary

Helminth infections and tuberculosis are two of the major health care problems worldwide and share a great deal of geographical overlap. Moreover, helminth infections are known to induce immune responses that are antagonistic to the protective immune responses elicited by *Mycobacterium tuberculosis*. Having previously demonstrated that helminth infections can profoundly alter protective T cell responses needed to control tuberculosis infection, we examined how *Strongyloides stercoralis* (Ss) infection influences B cell responses in latent tuberculosis infection (LTBI) in the context of co-infection and showed the Ss infection is associated with dramatic alterations in mycobacterial-specific IgG and IgM responses and levels of B cells and their growth factors BAFF and APRIL. These alterations in B cell responses could have implications for vaccine-induced immune responses to tuberculosis in helminth—endemic countries.

## Introduction

Helminth infections are powerful modulators of the immune response and typically elicit both Type 2 and regulatory cytokine responses [1,2]. Helminths can influence the host immune response to co-existent infections because of their propensity to establish longstanding, persistent infections that in turn can modulate host immunity [3]. For example, helminth infections are known to modulate the immune response to *Mycobacterium tuberculosis* (Mtb) in a variety of ways [4] including: 1) the down modulation of Th1 responses with diminished production of the cytokines IFNγ, TNFα and IL-2 [5,6,7]; 2) the down regulation of the Th17 (IL-17A, IL-17F and IL-22) response [5,6,7]; and 3) the induction of regulatory T cell responses [8].

While the T cell-mediated response is the cornerstone of the protective immune response to Mtb, recent evidence suggests that B cells can also play an important role [9,10]. Thus, human studies have demonstrated that antibodies in LTBI are functionally more competent than antibodies in those with active TB [11,12]. Moreover, active TB is characterized by altered levels of the B cell growth factors, BAFF and APRIL [13], that are crucial factors for peripheral B cell survival and antibody production [14]. In addition, those with active pulmonary tuberculosis (TB) are also known to have a dysfunctional circulating B cell compartment that can be reset following successful TB treatment [15].

Since helminth infections are also known to influence B cell survival and function [1], we postulated that helminth infections could affect Mtb-specific B cell responses in LTBI. We, therefore, sought to examine the B cell arm of the immune response in LTBI and how it is influenced by the presence of *Strongyloides stercoralis*, an intestinal helminth known to infect about 50–100 million people worldwide [16]. In so doing, we demonstrate that *S*. *stercoralis* infection is associated with alterations in the levels of Mtb–specific IgM and IgG, levels of BAFF and APRIL, and the number of B cells (and their component subsets) in LTBI and that most of these changes are reversible following anthelmintic therapy.

## Materials and methods

### Ethics statement

All individuals were examined as part of a natural history study protocol (12IN073) approved by Institutional Review Boards of the National Institute of Allergy and Infectious Diseases (USA) and the National Institute for Research in Tuberculosis (India). Informed written consent was obtained from all participants.

### Study population

We studied 132 individuals in Tamil Nadu, South India; 44 with LTBI and clinically asymptomatic *S*. *stercoralis* infection (hereafter LTBI/Ss), 44 with LTBI only (hereafter LTBI) and 44 with *S*. *stercoralis* infection alone (hereafter Ss) (Table 1). None had previous anthelmintic treatment nor HIV. Follow up was performed at 6 months following recruitment and treatment.

**Table 1:** Baseline demographics of study population.

Study Demographics	LTBI/Ss	LTBI	Ss
Number	n = 44	n = 44	n = 44
Sex (Male/Female)	26/18	27/17	25/19
Median age (range)	42 (22–64)	42 (24–60)	39 (20–61)
NIE ELISA	Positive	Negative	Positive
Quantiferon in Tube Gold	Positive	Positive	Negative

Those with LTBI were clinically asymptomatic with a positive QuantiFERON Gold-in-tube tests and normal chest radiographs. Active TB was excluded by sputum smear negativity. Ss infection was diagnosed by the presence of IgG antibodies to the recombinant NIE antigen as described previously [17,18]. None of the study population had other intestinal helminths (based on stool microscopy). All LTBI/Ss and Ss individuals were treated with single doses of ivermectin (12mg) and albendazole (400 mg) and follow–up blood draws from LTBI/Ss individuals were obtained six months later. Treated individuals were Ss infection negative by stool microscopy at six months post–treatment. All LTBI alone individuals were anti- Ss-NIE negative and negative for other intestinal helminths.

### Ex vivo analysis

Leukocyte counts and differentials were performed on all individuals using an AcT5 Diff hematology analyzer (Beckman Coulter, Brea, CA, USA). Whole blood was used for ex vivo phenotyping. Briefly, 250μl aliquots of whole blood was added to a cocktail of monoclonal antibodies specific for B cell subtypes and memory markers. B cell phenotyping was performed using antibodies directed against CD45-PerCP (clone 2D1, BD), CD19-Pacific Blue (clone H1B19; Biolegend, San Diego, CA, USA) CD27-APC-Cy7 (clone M-T271; BD), CD21-FITC (clone B-ly4; BD) CD20-PE (clone 2H7; BD) and CD10-APC (clone H110a; BD). Naive B cells were classified as CD45^+^ CD19^+^ CD21^+^ CD27^-^; classical memory B cells as CD45^+^ CD19^+^ CD21^+^ CD27^+^; activated memory B cells as CD45^+^ CD19^+^ CD21^-^ CD27^+^; atypical memory B cells as CD45^+^ CD19^+^ CD21^-^CD27^-^; immature B cells as CD45^+^ CD19^+^ CD21^+^ CD10^+^; and plasma cells as CD45^+^ CD19^+^ CD21^-^ CD20^-^ [19,20]). Following 30 min of incubation at room temperature, erythrocytes were lysed using 2 ml of FACS lysing solution (BD Biosciences, San Jose, CA, USA), and cells were washed twice with 2 ml of 1XPBS and suspended in 200 μl of PBS (Lonza, Walkersville, MD, USA). Eight- color flow cytometry was performed on a FACS Canto II flow cytometer with FACSDIVA software, version 6 (Becton Dickinson, Franklin Lakes, NJ, USA). The gating was set by forward and side scatter, and 100,000 gated events were acquired. Data were collected and analyzed using FLOW JO (TreeStar, Ashland, OR, USA). Leukocytes were gated using CD45 expression versus side scatter. Absolute counts of the subpopulations were calculated from flow cytometry and hematology data. A representative flow cytometry plot showing the gating strategies for B cell subsets is shown in the S1 Fig.

### Measurement of BAFF and APRIL

Plasma levels of BAFF (B cell activating factor) and APRIL (A proliferation-inducing ligand) (R&D Systems, Minneapolis, MN, USA) were measured using ELISA kits, according to the manufacturer's instructions.

### Measurement of IgM and IgG levels

Plasma levels of human TB antibody IgM and IgG (CUSABIO, College Park, MD, USA) were measured using ELISA kits, according to the manufacturer's instructions. The TB antigens used in the kit include both membrane and secreted antigens from Mtb H37Rv. The values are expressed as OD units.

### Statistical analysis

Data analyses were performed using GraphPad PRISM 6 (GraphPad Software, Inc., San Diego, CA, USA). Geometric means (GM) were used for measurements of central tendency. Statistically significant differences were analyzed using the nonparametric Mann-Whitney U test and Wilcoxon matched pair test. Multiple comparisons were corrected using the Holm’s correction. Correlations were calculated by the Spearman rank correlation test.

## Results

### Study population characteristics

The baseline demographics of the study population are shown in Table 1. As can be seen, there were no differences in age or sex between the groups. As expected, all of the individuals in the LTBI/Ss and Ss groups had IgG antibodies to the NIE antigen, while those in the LTBI (only) group did not have IgG antibodies to NIE. Similarly, those in the LTBI/Ss and LTBI groups were positive by QuantiFERON in–tube testing, indicative of latent *M*. *tuberculosis* infection, whereas those in the Ss group were not. The baseline hematological characteristics of the study populations are shown in Table 2. As can be seen, compared to the LTBI group, those with LTBI/Ss or Ss had significantly higher eosinophil and basophil counts. No significant differences in the other hematological parameters were observed.

**Table 2:** Baseline hematological parameters of study population.

Haematology profile	LTBI/Ss	LTBI	Ss	p Value
Red blood cell count, x10^6^/ul	4.8	4.89	4.5 (3.5–6.06)	NS
	(4.1–5.86)	(4.02–5.8)		
White blood cell count, x10^3^cells/ul	7,790	8,070	9,400 (5,800–16,900)	NS
	(5,100–13,300)	(5,200–11,900)		
Lymphocyte count, cells/ml	2,340	2,580	2,515 (1,553–3,711)	NS
	(1,000–3,750)	(1,610–4,110)		
Neutrophil count, cells/ml	3,770	4,350	4,803 (3,111–11,069)	NS
	(1,870–7,400)	(2,550–8,610)		
Monocyte count, cells/ml	560	590	662 (378–1,000)	NS
	(270–890)	(280–1,090)		
Eosinophil count, cells/ml	640	400	800 (102–3,519)	p = 0.0001
	(120–2,930)	(110–1,210)		
Basophil count, cells/ml	90	60	88 (12–306)	p<0.0001
	(40–330)	(10–380)		

### LTBI/SS co-infection is associated with diminished Mtb-specific IgM and IgG and BAFF and APRIL levels

**Fig 1:**
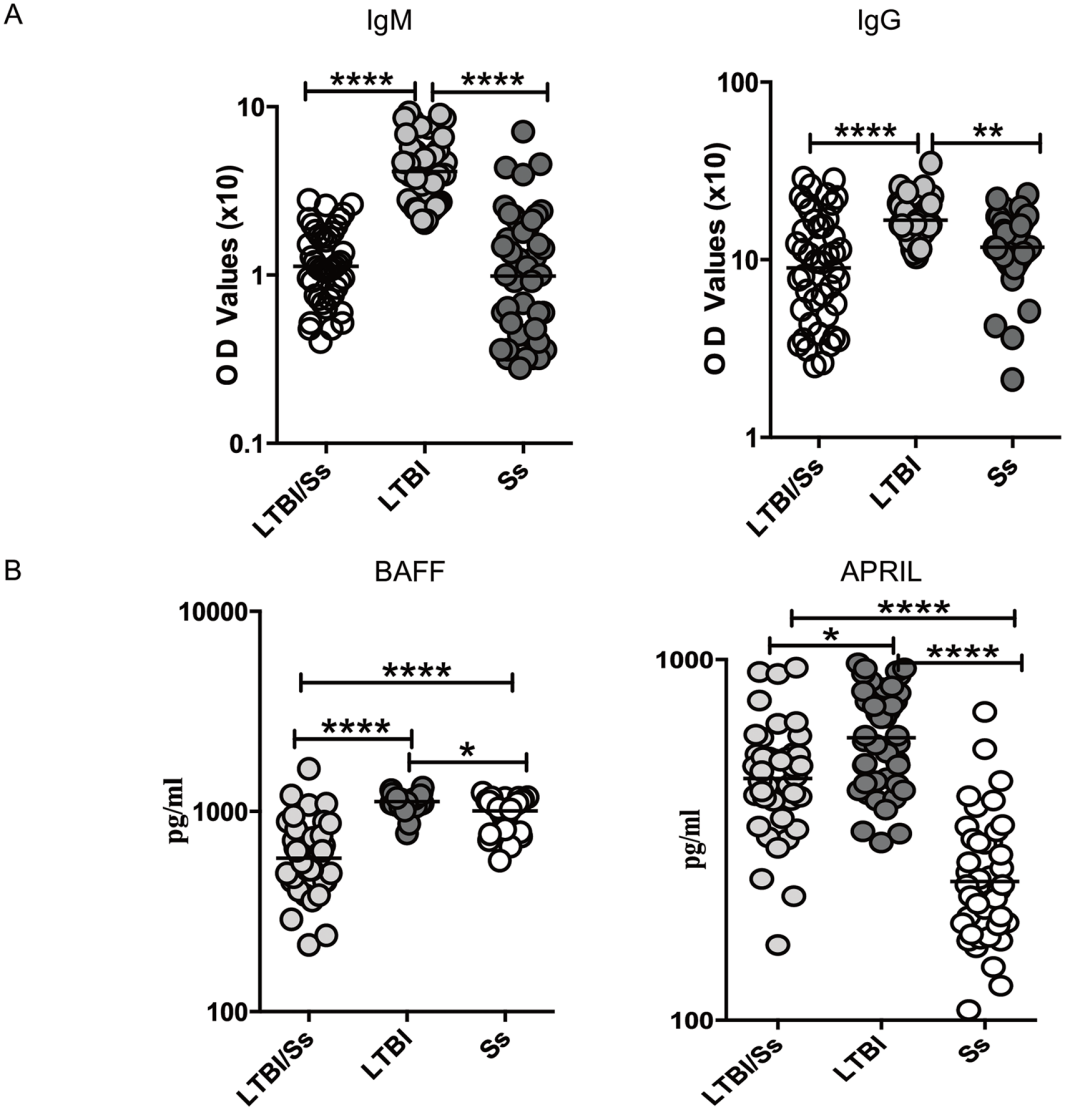
Strongyloides infection is associated with diminished systemic levels of IgM and IgG as well as BAFF and APRIL in latent tuberculosis. (A) The systemic levels of Mtb-specific IgM and IgG were measured in LTBI with (LTBI/Ss, n = 44) or without Ss co-infection (LTBI, n = 44) and in Ss infection only (Ss, n = 44). (B) The systemic levels of BAFF and APRIL were measured in LTBI with (LTBI/Ss, n = 44) or without Ss co-infection (LTBI, n = 44) and in Ss infection only (Ss, n = 44). The results are shown as scatter plots with each circle representing a single individual and the bar representing the GM. P values were calculated using the Mann-Whitney test with Holm's correction for multiple comparisons (* p<0.05, ** p<0.01, *** p<0.001, **** p<0.0001).

To characterize the antibody responses in LTBI/Ss co-infection, we first measured the levels of Mtb–specific IgM and IgG in LTBI/Ss and compared these to levels in LTBI or Ss. As shown in Fig 1A, the circulating levels of Mtb–specific IgM (GM of 0.11U) and IgG (GM of 0.89 U) in LTBI/Ss were significantly lower than in LTBI (GM IgM of 0.4 U and IgG of 1.67 U), but were no different from those in Ss (GM IgM 0.09 U and GM IgG of 1.18 U). This suggests that the coincident Ss infection in LTBI/Ss is associated with a reduction in the levels of Mtb-specific antibodies to those seen in Ss alone.

When the circulating levels of the B cell growth/differentiation factors BAFF and APRIL were measured in the 3 groups (Fig 1B), the systemic levels of BAFF (GM of 584.6 pg/ml in LTBI/Ss vs. 1118 pg/ml in LTBI and 1007 pg/ml in Ss) and APRIL (GM of 468.4 pg/ml in LTBI/Ss vs. 607.9 pg/ml in LTBI and 242.4 pg/ml in Ss) were significantly lower in LTBI/Ss group compared to those in the in LTBI and Ss groups.

### LTBI/Ss co-infection is associated with alterations in B cell numbers

**Fig 2:**
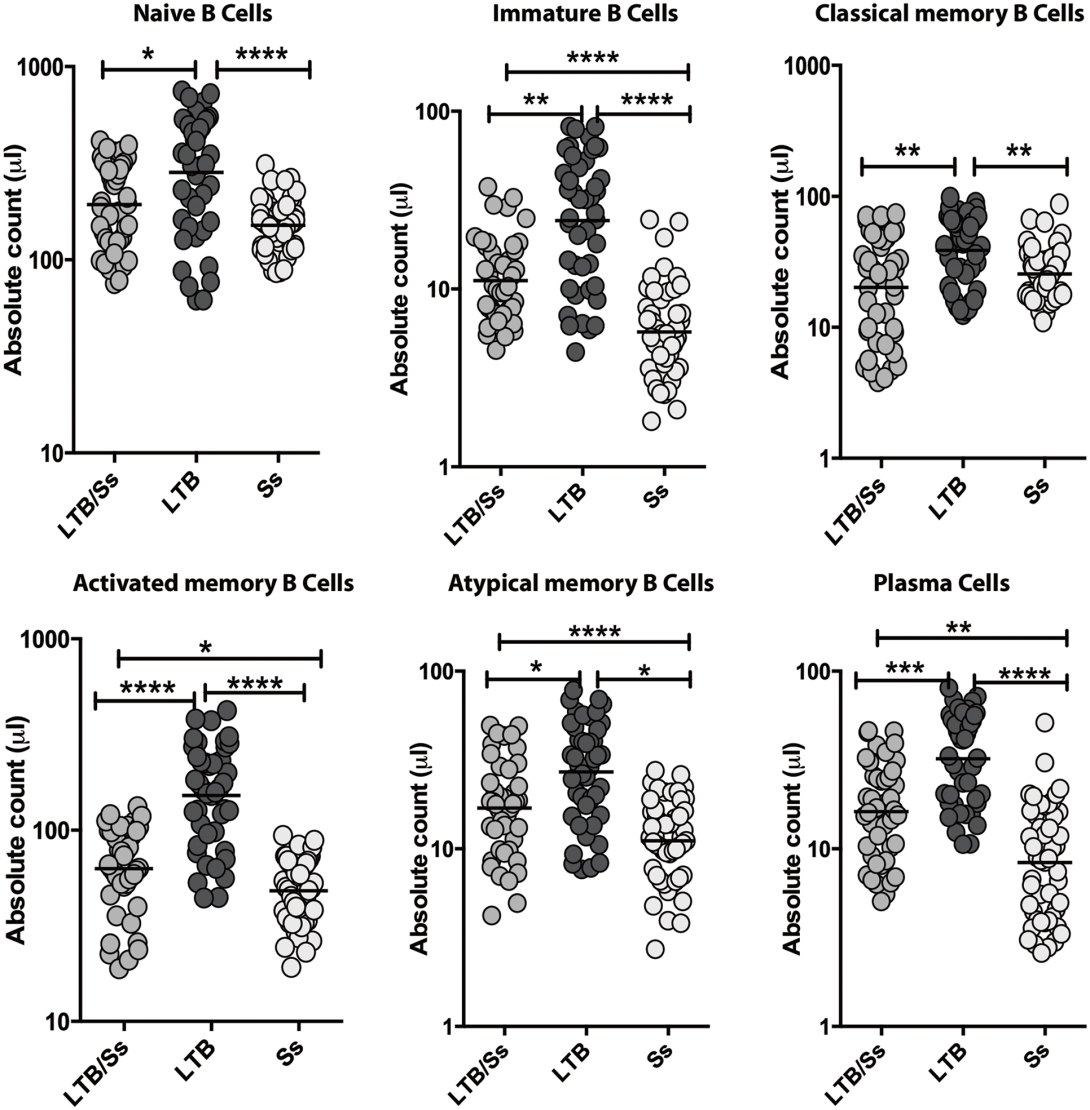
Strongyloides infection is associated with diminished absolute numbers of B cell subsets in latent tuberculosis. The absolute numbers of cells in each of the B cell subsets were measured in LTBI with (LTBI/Ss, n = 44) or without Ss co-infection (LTBI, n = 44) and in Ss infection only (Ss, n = 44). The results are shown as scatter plots with each circle representing a single individual and the bar representing the GM. P values were calculated using the Mann-Whitney test with Holm's correction for multiple comparisons (* p<0.05, ** p<0.01, *** p<0.001, **** p<0.0001).

To examine the *ex vivo* B cell (and B cell subset) phenotype LTBI/Ss co-infection, we analyzed the absolute numbers of each of the important B cell subsets in the 3 groups. As shown in Fig 2, the absolute numbers of naïve B cells and classical memory B cells were significantly lower in the LTBI/Ss group compared to the LTBI group. The absolute numbers of immature B cells, activated memory B cells, atypical memory B cells and plasma cells were significantly also lower in LTBI/Ss when compared to LTBI, but they were also significantly lower than the levels seen in Ss.

### Mtb–specific IgM and IgG levels are correlated with BAFF and APRIL levels, and numbers of memory B cells and plasma cells

**Fig 3:**
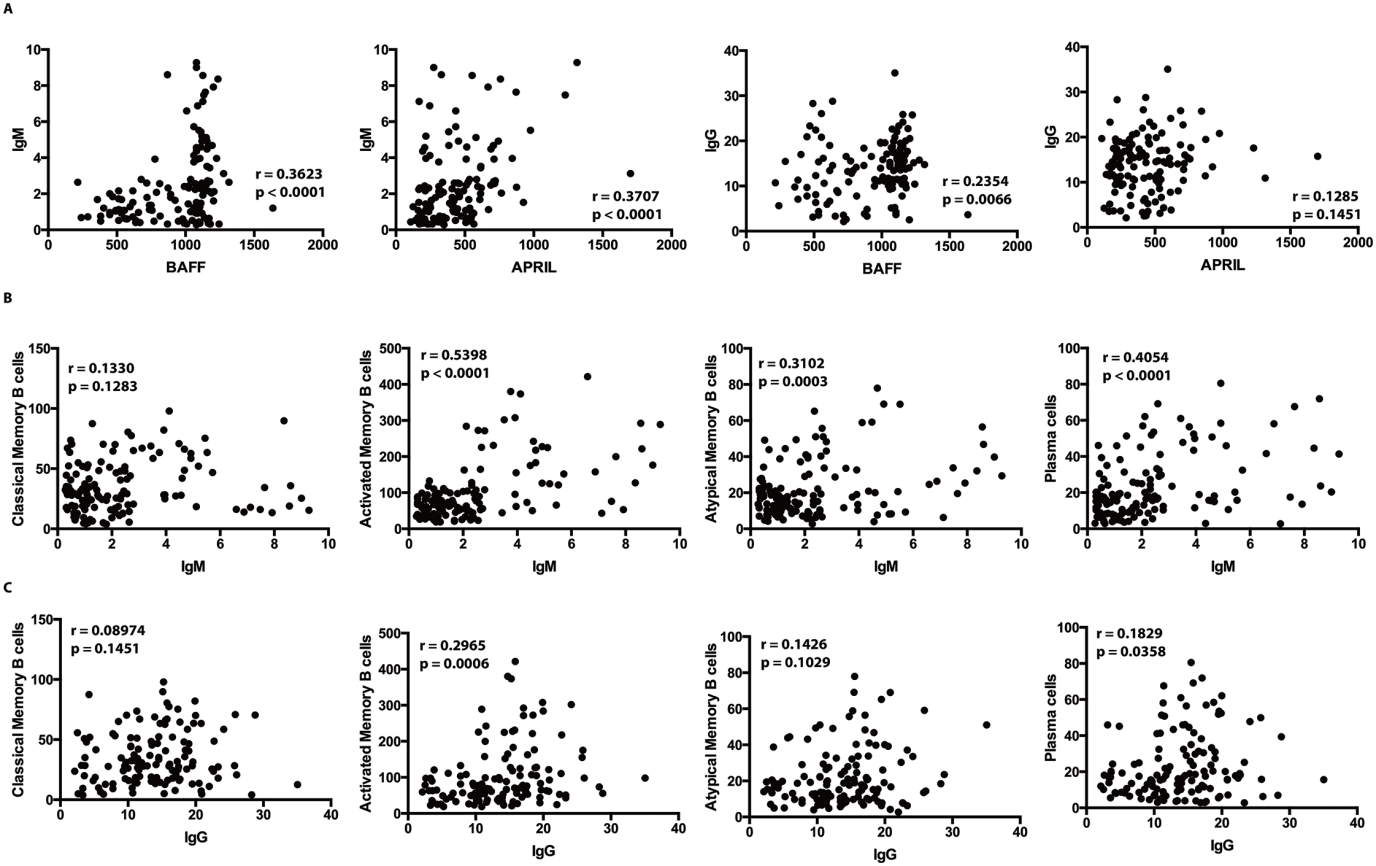
Circulating levels of Mtb-specific IgM and IgG correlate with BAFF/ APRIL and memory B cell subsets and plasma cells in LTBI/Ss and LTBI individuals. (A) The Mtb-specific IgM and IgG levels were correlated with the systemic levels of BAFF and APRIL in LTBI/Ss and LTBI (n = 88). (B) The Mtb-specific IgM levels were correlated with the absolute numbers of B cell subsets in LTBI/Ss and LTBI individuals together (n = 88). (C) The Mtb-specific IgG levels were correlated with the absolute numbers of B cell subsets in LTBI/Ss and LTBI individuals together (n = 88). The results are shown as scatter plots with each circle representing a single individual and the bar representing the geometric mean. P values were calculated using the Spearman Rank Correlation test.

Next, we performed correlation analyses between the levels of IgM and IgG with those of BAFF and APRIL and with the numbers of certain B cell subsets. As shown in Fig 3A, the circulating levels of Mtb–specific IgM showed a significant positive relationship with the systemic levels of BAFF and APRIL; the circulating levels of IgG only showed a significant relationship with BAFF (but not APRIL) levels. As shown in Fig 3B, the levels of IgM exhibited a significant positive relationship with numbers of activated and atypical memory B cells as well as plasma cells. Finally, as shown in Fig 3C, the circulating levels of IgG were positively associated with activated memory B cells and plasma cells. These data suggest that both IgM and IgG levels reflect levels of B cell growth factors and B cell memory subsets / plasma cells in LTBI/Ss coinfection.

### Anthelmintic therapy significantly increases Mtb–specific IgM and IgG levels and certain B cell subset numbers in LTBI/Ss co-infection

**Fig 4:**
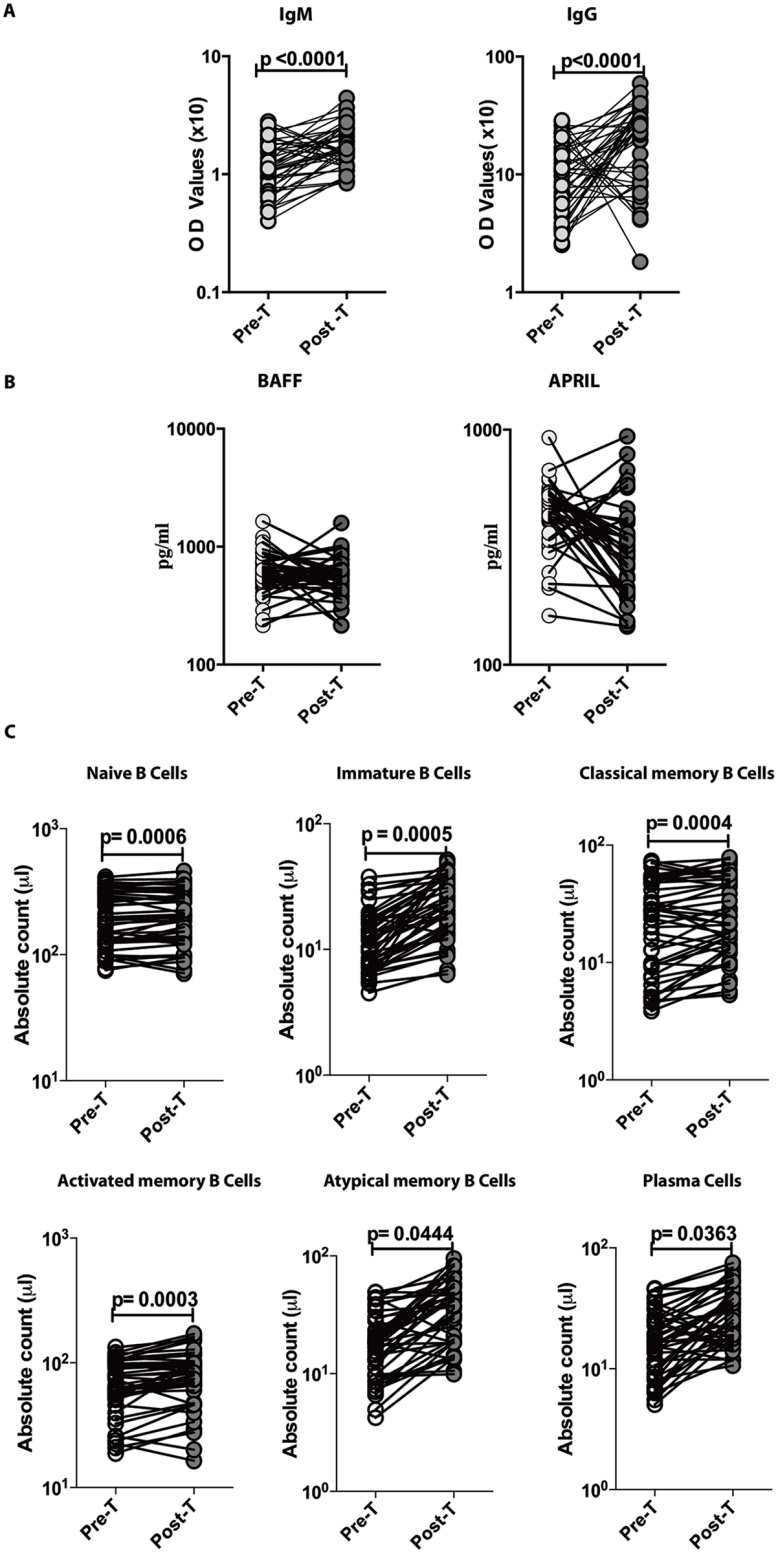
Anthelmintic treatment modifies the IgM and IgG levels as well as B cell subset numbers in LTBI/Ss co-infection. The levels of Mtb-specific IgM and IgG (Panel A), of BAFF and APRIL (Panel B) and the numbers of various B cell subsets (Panel C) were measured in in LTBI/Ss before (pre-T) and 6 months after (post-T) anthelmintic therapy. The data are shown as line diagrams with each line representing a single individual. P values were calculated using the Wilcoxon signed rank test with Holm's correction for multiple comparisons.

To examine whether the modulation of B cell responses in LTBI/Ss co-infection is reversible following anthelmintic therapy, we measured the levels of Mtb–specific IgM and IgG, the circulating levels of BAFF and APRIL and the numbers of various B cell subsets in LTBI/Ss individuals six months following anthelmintic treatment. As shown in Fig 4A, the circulating levels of Mtb–specific IgM and IgG levels increased significantly six months following anthelmintic treatment whereas there were no significant changes in levels of BAFF and APRIL (Fig 4B) in the LTBI/Ss group. Finally, as seen in Fig 4C, the absolute numbers of naïve B cells, immature B cells, classical memory B cells, activated memory B cells, atypical memory B cells and plasma cells were all significantly increased following treatment.

## Discussion

Infection caused by Mtb induces a strong humoral response in humans [10]. Although, T cell responses are considered the main driver of protective immunity, antibodies, may in part, contribute to protective immunity as well [9,10]. Thus, human antibodies have been shown to exert inhibitory activity against the growth of Mtb and to neutralize certain mycobacterial antigens (including virulence factor associated-antigens) that play important roles in host infection [12,21]. Based on an antibody profiling approach, it has also been demonstrated that in LTBI, there are distinct antibody profiles compared to the antibody profiles in those with active TB and that these antibodies can promote processes pivotal in host immunity, including enhanced phagolyosomal maturation, inflammasome activation and macrophage killing of intracellular Mtb [11]. In addition, antibodies, plasma cells and Fc receptor-bearing cells are abundant in TB granulomas [22,23] and antibodies against Mtb lipoarabinomannan induce increased bacterial opsonization and restrict growth [21,24]. Finally, mice lacking B cells or the ability to secrete antibodies are more susceptible to Mtb infection [25,26,27].

We tested the hypothesis that helminth co-infection can alter B cell responses in LTBI. Our data clearly reveal that Ss co-infection is associated with major effects on three different arms of the humoral immune response–antibody production, B cell growth factor levels and absolute numbers of B cells among the various subsets. Our data clearly illustrate that Ss co-infection is associated with significant modulation of the systemic levels of Mtb-specific IgM and IgG antibodies in the context of LTBI. IgM and IgG have the ability to opsonize antigens for complement mediated clearance, induce FcR mediated phagocytosis, direct anti-microbial activity by engagement of Fc receptors and augment cell mediated immune responses [28,29]. Therefore, the diminished levels of IgM and IgG in LTBI/Ss could potentially have detrimental effects in the immune response to TB. Interestingly, our post-treatment data also confirm a direct association of helminth infections on the modulation of B cell function in TB as the diminished levels of both IgM and IgG increased following successful anthelmintic treatment.

Our data also reveal important associations of Ss infection with BAFF and APRIL levels in LTBI/Ss. BAFF and APRIL are TNF-like cytokines that support the survival and differentiation of B cells [14]. BAFF is known to support naïve B cell survival and influences the development of other B cell subsets [14,30]. During antigen activation, BAFF upregulates TLR expression, promotes B cell survival and in collaboration with other cytokines, costimulatory signals, or TLR signals, promotes antibody class switching [30,31]. Moreover, BAFF in conjunction with inflammatory cytokines causes the induction of memory B cell differentiation into plasma cells [30,31]. APRIL is known to mainly function by amplifying the effects of BAFF on B cells [30,31]. Thus, BAFF and APRIL can profoundly influence B cell function; our data suggest that Ss infection is associated with significant modulation of their circulating levels.

Studies examining peripheral B cell numbers have suggested that B cell numbers are decreased in active TB compared to LTBI [15,32,33], a decrease that can normalize following definitive anti-tuberculous treatment [15]. Thus, LTBI appears to be characterized by higher numbers of different B cell subsets, and our data suggest a significant reduction in these numbers is associated with concomitant Ss infection. These results suggest a significant compromise in B cell distribution in the periphery. Combined with the finding of Ss-associated changes in absolute numbers of certain B cell subsets that are associated with changes in Mtb-specific IgM and IgG levels, our data indicate that Ss infection is associated with impaired functional responses of B cells.

In summary, our study has demonstrated clearly that Ss infections are associated with altered B cell responses and B cell subset numbers in the context of LTBI coinfection. While our study does not prove causation, it does provide evidence of a significant association of Ss infection with modulation of B cell function. With increasing data supporting a role of antibodies in protective immune responses to TB, our data add to the growing list of immunological mechanisms by which co-existent helminth infections can modulate responses in LTBI. They also suggest that treatment of helminth infection would make for a prudent first step in the conduct of TB vaccine trials in countries endemic for both TB and helminths.

## Supporting information

S1 Fig Gating strategy for B cell subsets. (A) A representative flow cytometry plot from an LTBI/Ss individual showing the gating strategy for naïve, immature, classical memory (CM), activated memory (AM), atypical memory (ATM), immature and plasma cells from CD45+ CD19+ cells. Naïve cells were classified as CD21+ CD27-; classical memory (CM) cells as CD21+ CD27+; activated memory (AM) cells as CD21- CD27+; Atypical memory (ATM) cell as CD21- CD27-; immature B cells as CD21+ CD10+; and plasma cells as CD21- CD27-.
(PDF)
