# Supplementary figures and images for: Modulation of Mycobacterium tuberculosis-specific humoral immune responses is associated with Strongyloides stercoralis co-infection
Source: PLoS Negl Trop Dis. 2017 May 1;11(5):e0005569. doi: 10.1371/journal.pntd.0005569 (PMC5426788; doi:10.1371/journal.pntd.0005569)

S. Figure. 1

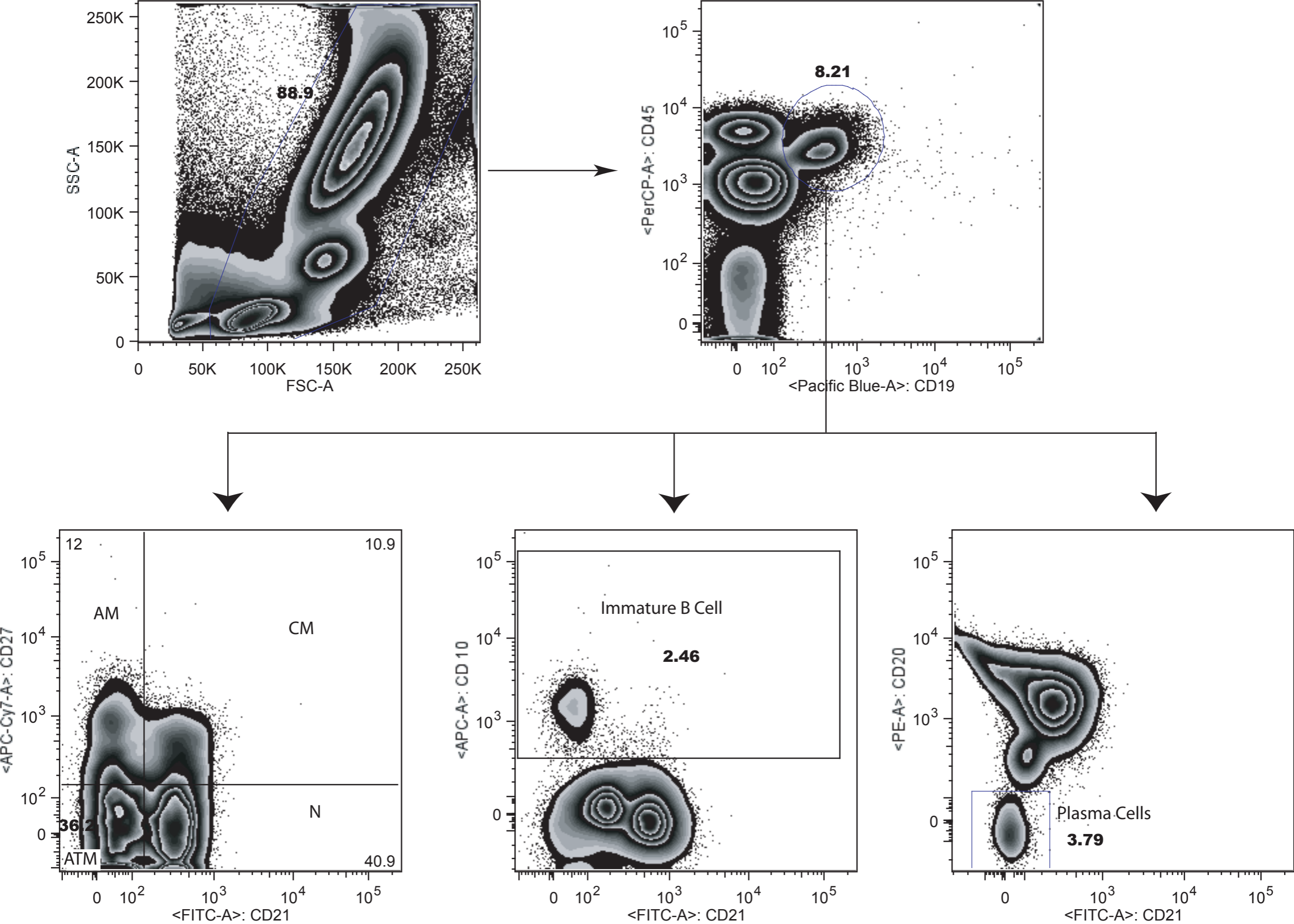

Supplement: S1 Fig — (A) A representative flow cytometry plot from an LTBI/Ss individual showing the gating strategy for naïve, immature, classical memory (CM), activated memory (AM), atypical memory (ATM), immature and plasma cells from CD45+ CD19+ cells. Naïve cells were classified as CD21+ CD27-; classical memory (CM) cells as CD21+ CD27+; activated memory (AM) cells as CD21- CD27+; Atypical memory (ATM) cell as CD21- CD27-; immature B cells as CD21+ CD10+; and plasma cells as CD21- CD27-. (PDF) [file pntd.0005569.s001.pdf]
